# What is the minimum response rate on patient-reported outcome measures needed to adequately evaluate total hip arthroplasties?

**DOI:** 10.1186/s12955-020-01628-1

**Published:** 2020-12-02

**Authors:** Yvette Pronk, Walter van der Weegen, Rein Vos, Justus-Martijn Brinkman, Ronald Johannes van Heerwaarden, Peter Pilot

**Affiliations:** 1 Research Department, Kliniek ViaSana, Hoogveldseweg 1, 5451 AA Mill, The Netherlands; 2 Department of Orthopaedic Surgery, Sint Anna Ziekenhuis, Bogardeind 2, 5664 EH Geldrop, The Netherlands; 3 Department of Methodology and Statistics, Maastricht University, P. Debyeplein 1, 6229 HA Maastricht, The Netherlands; 4 Department of Orthopaedic Surgery, Kliniek ViaSana, Hoogveldseweg 1, 5451 AA Mill, The Netherlands; 5 Stichting IMA, Kanaaldijk 10, 6116 AD Roosteren, The Netherlands

**Keywords:** Patient-reported outcome measures, Response rate, Total hip arthroplasty

## Abstract

**Background:**

Unknown is which response rate on patient-reported outcome measures (PROMs) is needed to both obtain an accurate outcome and ensure generalizability in evaluating total hip arthroplasty (THA) procedures. Without an evidence based minimum response rate (MRR) on THA PROMs, it is possible that hospitals report invalid patient-reported outcomes (PROs) due to a too low response rate. Alternatively, hospitals may invest too much in achieving an unnecessary high response rate. The aim of this study is to gain an insight into the MRR on PROMs needed to adequately evaluate THA procedures from a clinical perspective.

**Methods:**

Retrospective study on prospective collected data of primary, elective THA procedures was performed. MRR was investigated for each PROM (NRS pain at rest, NRS pain during activity, EQ-5D-3L, HOOS-PS, anchor function, OHS, anchor pain and NRS satisfaction) separately to calculate the primary outcome: MRR for the THA PROMs set. MRR on a PROM needed to have (condition 1.) similar PRO change score (3 month score minus preoperative score) including confidence interval, (condition 2.) maintaining the influence of each change score predictor and (condition 3.) equal distribution of each predictor, as those of a 100% PROM response rate group. Per PROM, a 100%-group was identified with all patients having the PRO change score. Randomly assessed groups of 90% till 10% response rate (in total 90 groups) were compared with the 100%-group. Linear mixed model analyses and linear regressions were executed.

**Results:**

The MRR for the THA PROMs set was 100% (range: 70–100% per PROM). The first condition resulted in a MRR of 60%, the second condition in a MRR of 100% and the third condition in a MRR of 10%.

**Conclusions:**

A 100% response rate on PROMs is needed in order to adequately evaluate THA procedures from a clinical perspective. All stakeholders using THA PROs should be aware that 100% of the THA patients should respond on both preoperative and 3 month postoperative PROMs. For now, taking the first step in improving evaluation of THA for quality control by achieving at least two of the three conditions of MRR, advised is to require a response rate on PROMs of 60% as the lower limit.

## Background

Total hip arthroplasty (THA) is performed to relieve pain, restore function and improve quality of life in patients with end-stage osteoarthritis. Patient-reported outcome measures (PROMs) gain insight into these results from a patients’ perspective. Nowadays, patient-reported outcomes (PROs) are collected on a large scale to evaluate THAs in hospitals and to compare THA health care between hospitals. PROs are seen as useful information to reflect on the clinical work executed as even on clinicians’ own executed care to improve patient care.

To draw valid conclusions on these evaluations, a certain response rate on PROMs is needed to both obtain an accurate outcome and ensure generalizability [1]. This minimum response rate (MRR), however, is unknown. The PROMs working group of International Society of Arthroplasty Registries (ISAR) advises a MRR of 60%. They mention that this is only based on the external difficulties to collect PROs that may be unrelated to survey logistics and the requirement of ≥ 60% for a survey study [2–4], however, without any further scientific evidence.

Since 2014, when THA PROs collection became mandatory in the Netherlands, huge differences are observed in response rate while comparing outcomes between Dutch hospitals; ranging from 10 to 100% preoperatively and from 2 to 95% at 3 months postoperatively [5, 6]. One might assume that these differences conceal a high risk of bias affecting the THA evaluation with PROs.

Achieving high PROMs response rate on multiple time points has proven to be even more challenging [7]. Even though automated collection systems are available, using these systems alone results in a moderate THA PROMs response rate on multiple time points (51%). A high response rate (> 90%) can be achieved with extra manual effort as sending paper questionnaires, but at an extra cost of around €6.0 per patient [7]. From a value-based health care perspective, it is debatable if these additional costs are justified as the MRR on PROMs for adequate evaluation of THA is unknown.

Without an evidence based MRR on THA PROMs, it is possible that hospitals report invalid PROs due to a too low response rate. Alternatively, hospitals may invest too much in achieving an unnecessary high response rate. Therefore, the aim of this study is to gain an insight into the MRR on PROMs needed to adequately evaluate THA procedures from a clinical perspective.

## Methods

A single centre retrospective study on prospective collected data from primary elective THA procedures was performed. THA procedures had been performed between March 2015 and December 2016 by three experienced high-volume orthopaedic surgeons in medium sized orthopaedic hospital (Kliniek ViaSana, Mill, the Netherlands). Patients were characterised by having an American Society of Anaesthesiologists (ASA) score of I or II, and a body mass index (BMI) of ≤ 35. Before each THA procedure, patients were informed, and asked to participate in PROs collection and to allow further scientific analysis using their anonymised data. All patients gave written informed consent. This study was approved by the district medical ethics committee (N18.156).

### PROs collection

The THA PROMs set included the mandatory PROMs as set out by the Dutch Orthopaedic Association (NOV) (Table 1) [4]. PROMs were collected preoperatively and at 3 months postoperatively with maximal effort to achieve 100% response rate [7]. PROs collection was preferably electronic using a digital, online, automated system (OnlinePROMs, Interactive Studios, Rosmalen, the Netherlands) with all questions obliged. In case patients were not or less able to handle a computer, paper questionnaires were sent by postal service. A maximum of three invitations to complete the questionnaires were sent. Patients with incomplete paper questionnaires were followed up by phone to complete all questionnaires [7]. Reasons for missing data were reported.

**Table 1:** Required and additional THA preoperative and 3 month postoperative PROMs [4]

THA PROMs set	PROM	Preoperative	3 months postoperative
Required PROMs	Pain by Numeric Rating Scale (NRS) – at rest (0 = no pain and 10 = unbearable pain)	✓	✓
	Pain by NRS – during activity (0 = no pain and 10 = unbearable pain)	✓	✓
	Quality of life by 3-level version of EuroQol 5 dimensions (EQ-5D-3L) (EQ VAS: 0 = worst imaginable health state and 100 = best imaginable health state; EQ-5D descriptive system: 0 = dead and 1 = healthy)	✓	✓
	Physical functioning by Hip disability and Osteoarthritis Outcome Score-Physical function Short-form (HOOS-PS) (0 = no difficulty and 100 = extreme difficulty) [8, 9]	✓	✓
	Anchor hip function (1 = very much deteriorated and 7 = very much improved)		✓
Additional PROMs	Hip specific function and pain by Oxford Hip Score (OHS) (0 = least difficulty and 48 = most difficulty) [10]	✓	✓
	Anchor hip pain (1 = very much deteriorated and 7 = very much improved)		✓
	Satisfaction by NRS (0 = very dissatisfied and 10 = very satisfied)		✓

*PROMs* patient-reported outcome measures, *THA* total hip arthroplasty

### Minimum response rate

The primary outcome was the MRR on the THA PROMs set, both required and additional PROMs, to adequately evaluate the results of THA. From a clinical perspective, evaluating the results of THA means evaluating the improvement patients made from before THA to a certain moment after THA. Minimal clinical important difference (MCID) does not yet exist for most THA PROMs, therefore, the change score was used as the best alternative. Three month change score (3 month score minus preoperative score) was utilized as this is a part of the Dutch PROMs indicator. Anchor questions regarding hip function and pain, and satisfaction question already measure a change, so these 3 month scores were seen as a change score.

The change score could be influenced by variables reported as predictors in previous studies: gender [11–13], age on the day of surgery [14–17], BMI [15, 18], Charnley score [11–13], comorbidity [12, 15] and anxiety [13, 19]. If a predictor influences the change score of the total THA patient group in this study (100% response rate group), this influence should be observed in smaller groups (lower response rate groups) as well to maintain the effect of the predictor on the change score. Furthermore, these predictors (for example gender) should exist of the same proportion (for example females and males) at a lower response rate to maintain a generalizable sample of the total THA patient group.

Therefore, the MRR was investigated for each PROM total- or subscore separately to calculate the MRR for the THA PROMs set. The MRR on a PROM needed to have (condition 1.) the similar change score including confidence interval (CI), (condition 2.) maintaining the influence of each change score predictor and (condition 3.) the equal distribution of each predictor as those of a 100% PROM response rate group. Regarding the THA PROMs set included, only quality of life measured using the 3-level version of EuroQol 5 dimensions (EQ-5D-3L) existed of two subscores instead of one totalscore (Table 1).

Besides PROs, patients characteristics including the known THA PROs predictors were assessed. Gender, age on the day of surgery (years), preoperative BMI (kg/m^2^), Charnley score (A, B1, B2, C), comorbidity (yes/no), ASA (I/II), osteoarthritis as diagnosis (yes/no) and complication (yes/no) were collected from the electronic patient records. Preoperative anxiety was measured using question 5 of the EQ-5D-3L of which answers 2 (moderately anxious or depressed) and 3 (extremely anxious or depressed) were grouped as having anxiety.

### Patient selection

A THA procedure was included when the patient signed informed consent form, was a valid responder and had a change score on one of the PROMs. A response was considered valid if the patient responded within the NOV selected time period (preoperative questionnaires: maximum 182 day before surgery; 3 month questionnaires: between 63 and 110 days after surgery) [4]. There were no exclusion criteria.

### Data analysis

Missing items were recalculated to complete the questionnaire if this was allowed according to the instrument-specific guidelines of the used questionnaires. To investigate if there was any difference between included and excluded THA procedures in patients characteristics including the predictors and preoperative PROs, independent t-tests or Mann–Whitney U tests for continuous variables were executed depending on the normal distribution of the data investigated using Shapiro–Wilk tests of normality and histograms, or Pearson’s chi-square or Fisher’s exact tests for categorical variables. Furthermore, variance patterns with respect to heteroscedasticity were investigated.

As missing PROs data are rarely MCAR and it was not sure if it was MAR of MNAR, to adopt an appropriate analytical strategy, three type of strategies were executed and results of the linear mix model analysis were compared: complete case analysis (MCAR or MAR), multiple impute missing data analysis with 200 imputations (MCAR or MAR) and sensitivity analyses (MNAR) [2]. These analyses were executed on the HOOS-PS which showed to have the most missing data. As no big deviations were found, complete case analysis was adapted in further analyses.

For each PROM total- or subscore, a 100%-group was identified with all included patients having the change score. Of this 100%-group, 10 times a random group of 90%, 10 times a random group of 80%, and so on for 70%, 60%, 50%, 40%, 30%, 20% and 10% were created (in total 91 groups). These groups were coded by the response rate and a random group number (for example 90,02). Linear mixed model analysis was used to assess differences in each PRO preoperatively and at 3 months postoperatively to investigate the change score of the 100%-group corrected by the 6 predictors. An unstructured covariance structure for the two repeated measures was used. This analysis method accounts for baseline differences and dependencies between repeated measures, and allowing unequal variances across groups. For PROs with one measurement (anchor questions hip function and pain, and satisfaction), this change score was analysed executing linear regression. P-values of the 6 predictors were checked. To compare the change score and the p-values of the predictors with all groups, in each group the same linear mixed model analysis or linear regression was performed. All group change scores with 95% CI or range were visualised in a graph (MRR condition 1). Regarding the predictors, defined was that 8 or more of the 10 groups of a certain response rate needed to have the same statistically significant or non-significant level as the 100%-group to be adequate (MRR condition 2).

To compare equal distribution of each predictor in each group to the 100%-group, Pearson’s chi-square or Fisher's exact tests were performed. Defined as adequate was that 8 or more of the 10 groups of a certain response rate had to have an equal distribution of a predictor (MRR condition 3). For this step, both age and BMI were transformed to categorical variables. Age was recorded to 5 groups: < 50 years, 50–59 years, 60–69 years, 70–79 years and ≥ 80 years. BMI was categorised to underweight (≤ 18.5), normal weight (> 18.5–25.0), overweight (> 25.0–30.0) and obesity (> 30.0–40.0) [20].

For all statistical analyses, an alpha of 0.05 was considered statistically significant and IBM SPSS Statistics 25.0 (IBM Corporation, U.S.) was used.

## Results

**Fig. 1:**
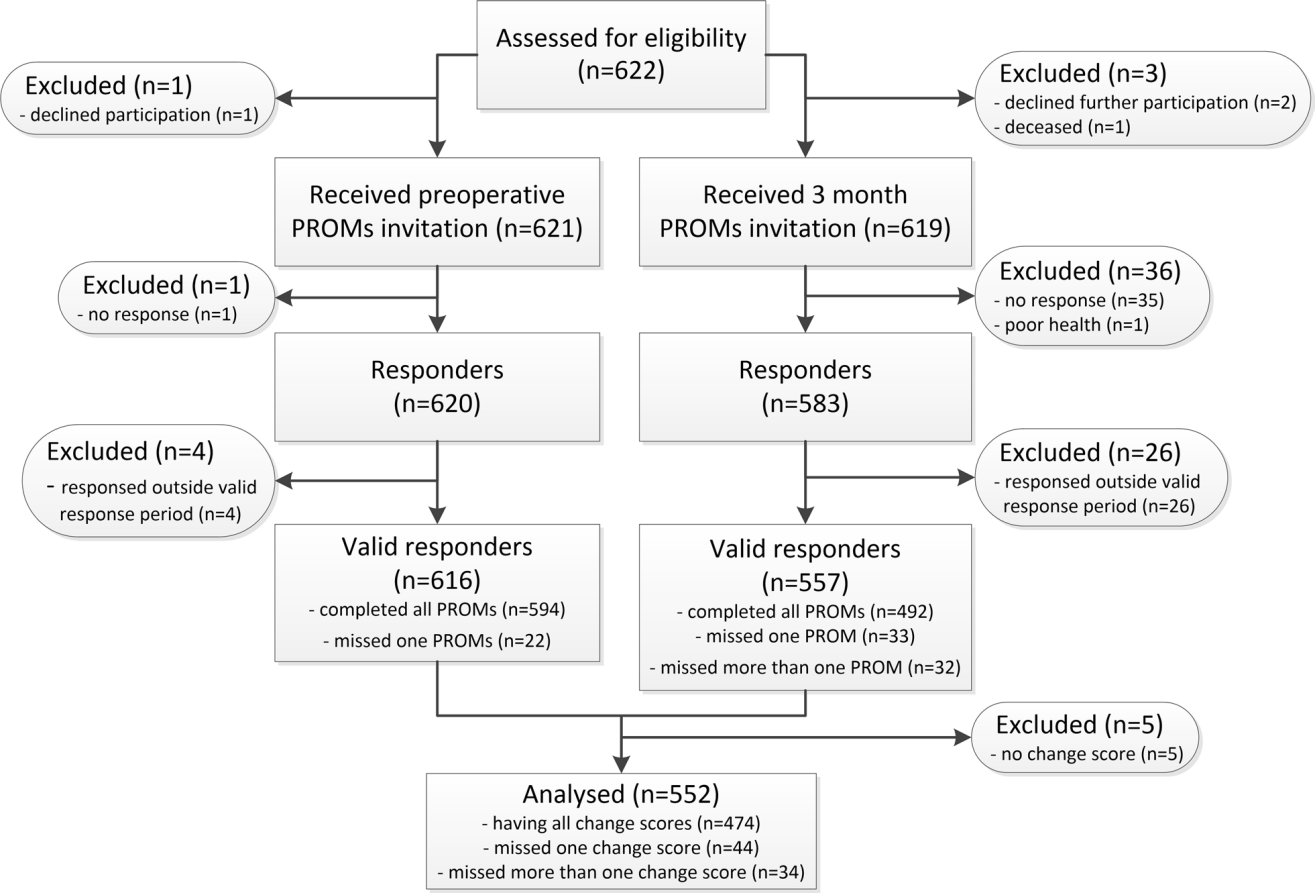
Study flowchart. n: number; PROMs: patient-reported outcome measures

During the study period 622 THA procedures (592 patients) were performed of which 616 (99.8%) were valid responders preoperatively and 557 (92.2%) at 3 months. Finally, 552 (88.8%) THA procedures were included. Main reasons for exclusion were no response preoperative and/or at 3 months postoperatively (n = 36 (5.8%)) and a response outside the valid preoperative and/or at 3 month postoperative response period (n = 30 (4.8%)). Of the 552 included THA procedures, 474 had all change scores available, the remaining 78 at least one (Fig. 1). No statistical significant differences regarding patients characteristics and preoperative PROs were found between the included and excluded THA procedures (Table 2).

**Table 2:** Patients characteristics and preoperative PROs of included and excluded THA procedures

Patients characteristics and preoperative PROs	Included THA procedures n = 552	Excluded THA procedures n = 70	*p* value
ASA classification (II; n (%))	284 (51%)	28 (40%)	0.071
Age on date of surgery (years; median (IQR))	66 (60–71)	65 (55–74)	0.805
BMI (kg/m^2^; median (IQR))	26.00 (23.90–28.41)	26.29 (24.48–28.13)	0.389
Gender (male; n (%))	209 (38%)	31 (44%)	0.298
Diagnosis (osteoarthritis; n (%))	486 (88%)	60 (86%)	0.575
Charnley score (n (%))			0.064
A—one hip joint affected	135 (24%)	15 (21%)	
B1—both hip joints affected	245 (44%)	23 (33%)	
B2—contralateral hip joint with a total hip prosthesis	110 (20%)	17 (24%)	
C—multiple joints affected	62 (11%)	15 (21%)	
Comorbidity (yes, n (%))	178 (32%)	23 (33%)	0.918
Anxiety (yes, n (%))	123 (22%)	20 (29%)	0.188
Preoperative NRS pain at rest (median (IQR))	6 (4–7)	5 (4–7)	0.543
Preoperative NRS pain during activity (median (IQR))	8 (7–9)	8 (7–9)	0.363
Preoperative EQ-5D descriptive system (median (IQR))	0.693 (0.310–0.775)	0.693 (0.335–0.775)	0.625
Preoperative EQ VAS (median (IQR))	80 (60–87)	77 (66–85)	0.960
Preoperative HOOS-PS (median (IQR))	46.1 (37.7–55.9)	50.8 (41.7–55.9)	0.341
Preoperative OHS (median (IQR))	24 (18–29)	24 (17–29)	0.454
Complication (yes, n (%))	33 (6%)	8 (11%)	0.118

*ASA* American Society of Anaesthesiologists, *BMI* body mass index, *EQ-5D descriptive system* EuroQol 5 dimensions descriptive system, *EQ VAS* EuroQol Visual Analogical Scale, *HOOS-PS* Hip disability and Osteoarthritis Outcome Score-Physical function Short-form, *NRS* numeric rating scale, *OHS* Oxford Hip Score, *PROs* patient-reported outcomes, *THA* total hip arthroplasty

### Missing data

Most of the 78 patients, who had not all change scores, had no HOOS-PS change score due to missing data in the HOOS-PS 3 month questionnaire (n = 59 (10.7%)) or had no EQ VAS change score due to missing data in the EQ VAS question at 3 months (n = 31 (5.6%)). Main reason for missing data on this HOOS-PS 3 month questionnaire was about the item running. Patients were advised not to run after THA surgery and the question asked to indicate the degree of difficulty experienced in performing this activity.

Different strategies for missing data were executed. Mixed model analysis with complete cases reported a mean HOOS-PS change score of -32.4 (CI: −34.1–−30.8) (n = 480), with multiple imputed missing data a mean of −32.5 (CI: −32.6–−32.4), with imputed worst scores a mean of −33.2 (CI: −34.9–−31.5) (n = 552) and with imputed best scores a mean of -29.1 (CI: −31.1–−27.1) (n = 552). Maximum difference between these strategies was 4.1 points for the change score resulted in a 2.1% difference on the HOOS-PS change score scale of −100 to 100. The CI ranged from 0.2 to 4.0 in size. Only in the analysis with imputed worst scores, the predictor anxiety was not a significant predictor (*p* = 0.053) and age was (*p* = 0.001). The estimate changes of the predictors were, however, similar in all analyses. Based on these small differences found, complete case analysis was adapted in further analyses.

### MRR for NRS pain at rest

**Fig. 2:**
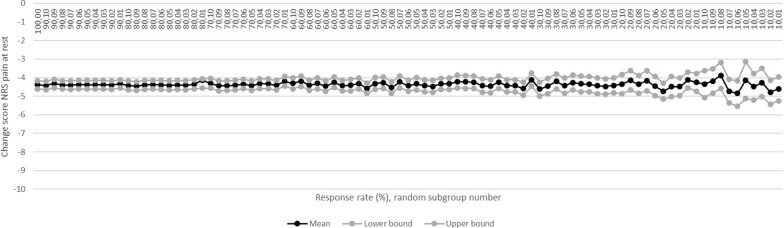
Mean NRS pain at rest change score per group. NRS: numeric rating scale

In the 100% NRS-pain-at-rest-group the mean change score was −4.4 (CI: −4.6–−4.2) (n = 551) which was no longer similar when the response rate dropped below 30%. Mean change score in the 20%-groups was −4.4 (CI: −4.8–−3.9). This score was similar and the CI was 2.3 times (230%) greater (0.9 versus 0.4) compared to the 100%-group (Fig. 2; condition 1). Gender (*p* = 0.001), comorbidity (*p* = 0.041), age (*p* = 0.002) and BMI (*p* = 0.018) were significant predictors in the 100%-group which remained down to and including the 60%, 100%, 60% and 100%-group respectively. Charnley score and anxiety remained no significant predictors down to and including the 10%-groups (condition 2). Equal distributions of all predictors were observed down to the 10%-groups inclusive compared to the 100%-group (Table 3; condition 3).

**Table 3:** Number of NRS pain at rest groups per response rate with predictors as significant predictor or equal distribution

	Charnley score		Gender		Comorbidity		Anxiety		Age		BMI	
	Significant predictor?	Equal distribution?	Significant predictor?	Equal distribution?	Significant predictor?	Equal distribution?	Significant predictor?	Equal distribution?	Significant predictor?	Equal distribution?	Significant predictor?	Equal distribution?
100% (p-value)	0.435	x	0.001	x	0.041	x	0.631	x	0.002	x	0.018	x
90% (n)	0	10	10	10	7	10	0	10	10	10	7	10
80% (n)	0	10	10	10	5	10	0	10	10	10	7	10
70% (n)	0	10	8	10	3	10	0	10	10	10	3	10
60% (n)	0	10	9	10	1	10	0	10	8	10	5	10
50% (n)	1	10	7	10	2	10	0	10	6	10	2	10
40% (n)	0	10	6	10	3	10	0	10	3	10	4	10
30% (n)	2	10	3	10	0	10	1	10	1	10	5	10
20% (n)	1	10	2	10	0	10	0	10	4	10	2	10
10% (n)	0	9	1	10	2	10	0	10	2	10	2	10

*BMI* body mass index

### MRR for NRS pain during activity

The mean change score of −5.4 (CI: −5.6–−5.2) (n = 551) found in the 100% NRS-pain-during-activity-group was observed down to and including the 30%-groups. In the 20%-groups, the mean change score was −5.4 (CI: −5.9–−4.9). Compared to the 100%-group, this score was similar and the CI was 2.5 times (250%) greater (1.0 versus 0.4) (Additional file 1, Fig. 1; condition 1). Gender (*p* = 0.000) and age (*p* = 0.000) were significant predictors for this change score in the 100%-group which remained down to and including the 40% and 60%-groups respectively. BMI remained a non-significant predictor down to the 100%-group. The other predictors stayed non-significant predictors in all groups (condition 2). Down to the 10%-groups inclusive, equal distribution of all predictors was found compared to the 100%-group (Additional file 1, Table 1; condition 3).

### MRR for EQ-5D-3L

#### EQ-5D descriptive system

The mean change score of 0.250 (CI: 0.225–0.274) in the 100% EQ-5D descriptive system group (n = 544) was observed down to and including the 30%-groups. The 20%-groups reported a mean change score of 0.249 (CI: 0.195–0.303). This score differed 0.001 points (0.4%) and the CI was 2.2 times (220%) greater (0.108 versus 0.049) compared to the 100%-group (Additional file 1, Fig. 2; condition 1). Regarding the significant predictors, gender (*p* = 0.001) was found to be a significant predictor down to the 50%-groups inclusive, anxiety (*p* = 0.000) to 10%, age (*p* = 0.004) to 80% and BMI (*p* = 0.019) to 100%. Comorbidity remained a non-significant predictor down to and including the 60%-groups (condition 2). All predictors were equal distributed down to the 10%-groups inclusive compared to the 100%-group (Additional file 1, Table 2; condition 3).

#### EQ VAS

The 100% EQ VAS group had a mean EQ VAS change score of 7.1 (CI: 5.3–8.8) (n = 521) and showed to remain similar down to and including the 40%-groups. Mean change score in the 30%-groups was 7.2 (CI: 4.0–10.5). Compared to the 100%-group, this score differed 0.1 point (1.4%) and the CI was 1.9 times (190%) greater (6.5 versus 3.5) (Additional file 1, Fig. 3; condition 1). Gender (*p* = 0.001), comorbidity (*p* = 0.003) and anxiety (*p* = 0.000) were significant predictors in the 100%-group and down to the 70%, 60% and 50%-groups inclusive respectively. The other predictors remained non-significant predictors in all groups (condition 2). Equal distribution was found down to and including the 10%-groups for all predictors compared to the 100%-group (Additional file 1, Table 3; condition 3).

**Fig. 3:**
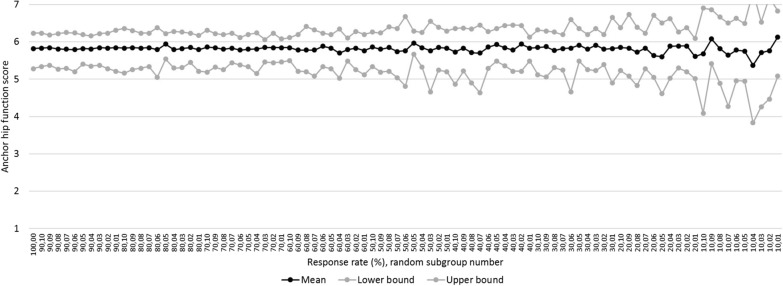
Mean anchor hip function score per group

### MRR for HOOS-PS

The mean change score of the 100% HOOS-PS group was −32.4 (CI: −34.1–−30.8) (n = 480) and found to be similar down to and including the 40%-groups. The 30%-groups reported a mean change score of −32.2 (CI: −35.1–−29.2). This score differed 0.2 points (0.6%) and the CI was 1.8 times (180%) greater (5.9 versus 3.3) compared to the 100%-group (Additional file 1, Fig. 4; condition 1). Significant predictors were gender (*p* = 0.000) and anxiety (*p* = 0.003) which both remained down to the 60%-groups inclusive. Charnley score and BMI stayed non-significant predictors down to the 60% and 90%-groups inclusive respectively (condition 2). All predictors were equally distributed down to and including the 10%-groups compared to the 100%-group (Additional file 1, Table 4; condition 3).

**Fig. 4:**
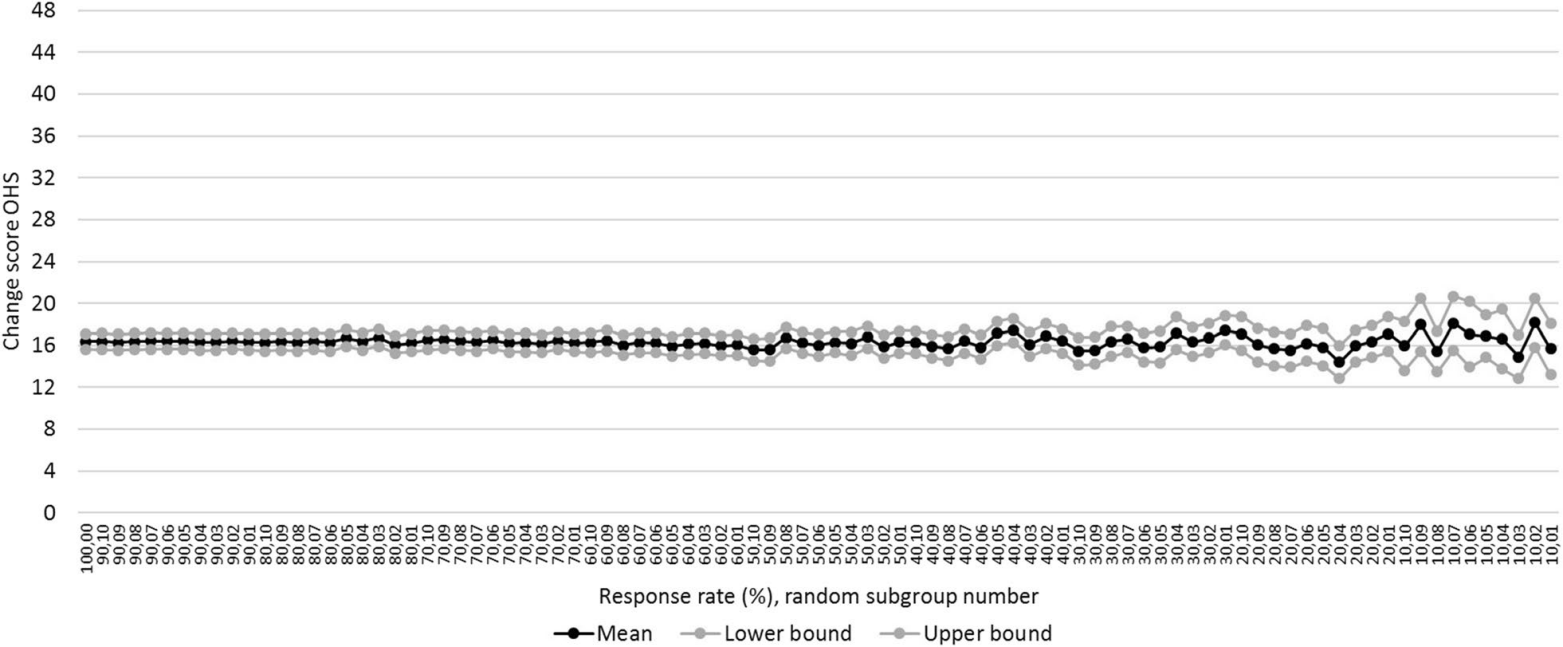
Mean OHS change score per group. OHS: Oxford Hip Score

**Table 4:** Number of anchor function groups per response rate with predictors as significant predictor or equal distribution

	Charnley score		Gender		Comorbidity		Anxiety		Age		BMI	
	Significant predictor?	Equal distribution?	Significant predictor?	Equal distribution?	Significant predictor?	Equal distribution?	Significant predictor?	Equal distribution?	Significant predictor?	Equal distribution?	Significant predictor?	Equal distribution?
100% (*p* value)	0.339	x	0.113	x	0.248	x	0.341	x	0.496	x	0.051	x
90% (n)	0	10	0	10	0	10	0	10	0	10	2	10
80% (n)	0	10	1	10	0	10	0	10	0	10	4	10
70% (n)	0	10	2	10	0	10	0	10	0	10	2	10
60% (n)	0	10	0	10	1	10	0	10	0	10	0	10
50% (n)	1	10	3	10	3	10	1	10	0	10	2	10
40% (n)	0	10	2	10	0	10	1	10	0	10	3	10
30% (n)	1	10	0	10	0	10	0	10	0	10	2	10
20% (n)	1	10	0	8	0	10	0	10	0	10	1	10
10% (n)	1	10	0	8	1	9	0	10	1	10	3	10

*BMI* body mass index

### MRR anchor hip function

The mean anchor hip function was 5.8 (CI: 5.3–6.2) in the 100%-group (n = 540) and showed to be similar down to and including the 60%-groups. Regarding the 50%-groups, the mean score was 5.8 (CI: 5.2–6.4). This score was similar and the CI was 1.3 times (133%) greater (1.2 vs. 0.9) compared to the 100%-group (Fig. 3; condition 1). In the 100%-group, there were no significant predictors which remained down to and including the 60%-groups for gender and for comorbidity, the 90%-groups for BMI and the 10%-groups for the other predictors (condition 2). Equal distribution was found in all predictors down to the 10%-groups inclusive compared to the 100%-group (Table 4; condition 3).

### MRR for OHS

In the 100% OHS group a mean change score of 16.4 (CI: 15.7–17.1) was found (n = 542) and observed to be similar down to and including the 30%-groups. The 20%-groups had a mean change score of 16.0 (CI: 14.4–17.6). Compared to the 100%-group, this score differed 0.4 points (2.4%) and the CI was 2.3 times (230%) greater (3.2 vs. 1.4) (Fig. 4; condition 1). Regarding the predictors, gender (*p* = 0.000), anxiety (*p* = 0.000), age (*p* = 0.016) and BMI (*p* = 0.001) were significant predictors in the 100%-group which remained down to the 50%, 30%, 100% and 50%-groups inclusive respectively. Both Charnley score and comorbidity stayed non-significant predictors (condition 2). Down to and including the 10%-groups, all predictors showed to have an equal distribution compared to the 100%-group (Table 5; condition 3).

**Table 5:** Number of OHS groups per response rate with predictors as significant predictor or equal distribution

	Charnley score		Gender		Comorbidity		Anxiety		Age		BMI	
	Significant predictor?	Equal distribution?	Significant predictor?	Equal distribution?	Significant predictor?	Equal distribution?	Significant predictor?	Equal distribution?	Significant predictor?	Equal distribution?	Significant predictor?	Equal distribution?
100% (*p* value)	0.306	x	0.000	x	0.234	x	0.000	x	0.016	x	0.001	x
90% (n)	0	10	10	10	0	10	10	10	6	10	10	10
80% (n)	0	10	10	10	0	10	10	10	8	10	10	10
70% (n)	0	10	10	10	1	10	10	10	4	10	9	10
60% (n)	1	10	9	10	1	10	10	10	6	10	8	10
50% (n)	1	10	9	10	0	10	10	9	4	10	9	10
40% (n)	1	10	7	10	0	10	10	10	5	10	4	10
30% (n)	0	10	6	10	0	10	10	10	2	10	3	10
20% (n)	1	10	7	10	0	10	2	9	2	10	3	10
10% (n)	0	10	1	10	1	10	1	10	1	9	1	10

*BMI* body mass index

### MRR for anchor hip pain

The 100% anchor hip pain group had a mean score of 6.2 (CI: 5.7–6.5) (n = 539) and showed to be similar down to and including the 50%-groups. The 40%-groups had a mean score of 6.2 (CI: 5.7–6.6). This score was similar and the CI was 1.1 times (110%) greater (0.9 versus 0.8) compared to the 100%-group (Additional file 1, Fig. 5; condition 1). Significant predictors of this score were gender (*p* = 0.040) and comorbidity (*p* = 0.022) in the 100%-group, both remaining significant down to the 100%-group inclusive. The other predictors stayed non-significant predictors in all groups (condition 2). Down to and including the 10%-groups, all predictors were equally distributed compared to the 100%-group (Additional file 1, Table 5; condition 3).

### MRR for satisfaction

The mean NRS satisfaction score in the 100%-group was 8.5 (CI: 7.5–9.3) (n = 537) and was observed to be similar down to and including the 60%-groups. The 50%-groups reported a mean score of 8.6 (CI: 7.5–9.4). This score differed 0.1 points (1.2%) and the CI was 1.1 (110%) greater (1.9 versus 1.8) compared to the 100%-group (Additional file 1, Fig. 6; condition 1). In the 100%-group, gender (*p* = 0.013) and BMI (*p* = 0.029) were significant predictors which stayed down to and including the 90% and 100%-group respectively. Age and the other predictors remained non-significant predictors down to the 30% and 100%-group inclusive respectively (condition 2). Compared to the 100%-group, equal distribution was found in all predictors down to the 10%-groups inclusive (Additional file 1, Table 6; condition 3).

**Table 6:** MRR for each THA PROM including per complied condition

THA PROMs set	PROM	1. Similar change score (%)	2. Maintaining influence of predictors (%)	3. Equal distribution of predictors (%)	MRR (%)
Required	NRS pain at rest	30	100	10	100
	NRS pain during activity	30	100	10	100
	EQ-5D-3L				
	EQ-5D descriptive system	30	100	10	100
	EQ VAS	40	70	10	70
	HOOS-PS	40	90	10	90
	Anchor hip function	60	90	10	90
Required set		60	100	10	100
Additional	OHS	30	100	10	100
	Anchor hip pain	50	100	10	100
	Satisfaction	60	100	10	100
Total set		60	100	10	100

*EQ-5D descriptive system* EuroQol 5 dimensions descriptive system, *EQ VAS* EuroQol Visual Analogue Scale, *HOOS-PS* Hip disability and Osteoarthritis Outcome Score-Physical function Short-form, *MRR* minimum response rate, *NRS* numeric rating scale, *OHS* Oxford Hip Score, *PROMs* patient-reported outcome measures, *THA* total hip arthroplasty

### MRR for THA PROMs set

To investigate the MRR of the THA PROMs set, summarized: condition 1 resulted in a MRR of 60% (30–60%) for both the total THA PROMs set as only the required THA PROMs set, condition 2 in a MRR of 100% (70–100%) respectively and condition 3 in a MRR of 10% (10–10%) respectively. MRR per PROM ranged from 70 to 100% (Table 6).

## Discussion

Gaining an insight into the response rate on PROMs needed to adequately evaluate THA procedures from a clinical perspective was the aim of this study. Results show that for the Dutch THA PROMs set a 100% (range: 70% to 100% per PROM) response rate is needed. It was not possible to lower this MRR of 100% due to not maintaining the influence of each change score predictor at a lower response rate (condition 2). Still measuring the similar change score (condition 1) resulted in a MRR of 60% and still maintaining equal distribution of each predictor (condition 3) in a MRR of 10%.

In many countries, PROs are measured routinely and incorporated into arthroplasty registers. PROs are evaluated in hospitals, compared between hospitals and even financial incentives are based on these outcomes. For each hospital as even for each clinician, PROs are seen as useful information to reflect on the clinical work executed to improve patient care. From a clinical perspective, for adequate evaluation of THA with PROs a response rate of 100% is needed, shown by the current study (Table 6). This means that 100% of the THA patients should respond on the preoperative PROMs as well as on the 3 month postoperative PROMs. However, it is impossible to achieve this in clinical practice. None of the hospitals reached a 100% response rate on THA PROMs preoperatively as well as postoperatively; mean reported response rate on both time points is 37% in the Dutch register and 79% in the Swedish register [6, 21].

A first step in improving THA evaluation with PROs from a clinical perspective for quality control can be made by achieving at least two of the three MRR conditions (Table 6). This results in a MRR of 60% as the lower limit of evaluating THA outcome using PROs meaning 60% of the patients should be a responder on the preoperative as well as on the 3 month postoperative PROMs. Advised is to discard PROs collected below 60% to prevent for both invalid in-hospital evaluation as for invalid comparisons between hospitals. As a consequence, to achieve the lower limit of 60%, ISAR should tighten up their MRR advice and hospitals should increase their response rates beyond 60% if they are not there yet.

Interestingly, to a certain extent lower response rates are acceptable provided that MCIDs are evaluated [22]. Comparison between PROs of patients with lumbar discectomy incorporated into the Swedish spine register with PROs of the same patient population of a single hospital showed significant different change scores in PROs, but all within the MCIDs [22]. It could be that in the present study the observed differences in change scores in lower response rate groups compared to the 100%-group are still within clinical relevant difference. However, yet no MCIDs or comparable values are available for most THA PROMs as even the best method to determine them [23, 24]. One study investigated and reported the 6 month OHS MCID at group level of around 11 points [25]. Comparing this with the results of the present study, MRR for OHS could be 10% instead of 30% (Fig. 4). The current study should be repeated when these MCIDs based on a golden standard method to determine them are known.

Although practice shows difficulties in achieving high response rates, response rates of > 80% are achievable in orthopaedic patients [7, 26–29]. It is even shown to be feasible to achieve > 90% response rate in busy orthopaedic hospitals, urban and rural, using a digital collection system without any major disruption to the clinical work flow [29]. As seen in the current study, ASA classification and Charnley score were almost significant predictors for being a responder or not. However, achieving high response rates depends more on the method in PROs collection chosen. Making PROs collection a part of routine care, using a PROMs digital administration station in the hospital and collecting via multiple sources (for example mail and email) are the keys to high response rates [7, 27–29]. In arthroplasty patients, a critical factor is making sure PROs are collected preoperatively as it results in a 3 times more chance of collecting the PROs 3 months after surgery and even a 15 times more chance at 12 months [30]. Maintaining high postoperative response rates is crucial as non-responding patients can introduce bias which results in incomparable PROs if the non-responders are different than the responders [28, 31] and missing data are not at random [32]. Therefore, it is advised that hospitals should take the winners in effort and costs in this method to at least reach the lower limit of 60% response rate.

For this first study tackling the methodological challenge in investigating the required response rate to ensure THA PROs could be used to adequately evaluate THA procedures from a clinical perspective, several assumptions had to be made to create a starting point in clarifying this issue. This study used the change scores at 3 months postoperatively (towards preoperative). Complexity exists as this study should be repeated for change scores at 12 and 24 months postoperatively towards preoperative and even at 12 and 24 months postoperatively towards 3 months postoperatively to have a more complete answer. Acquiring a complete PROMs dataset including also 12 and 24 months results is even more challenging than a dataset including only preoperative and 3 months results. The method chosen for this challenge was considered as the only option due to unequal variances and unknown MCIDs. Future research should investigate if the MCIDs instead of change scores remain similar in lower response rates when these MCIDs are available. Another assumption made was that all three conditions are of the same value. Future research should investigate if this is indeed the case. Case-mix is important in investigating MRR. Based on previous literature, six predictors were incorporated in all three conditions besides only correcting for them to adjust the change score in condition 1. As case-mix is another methodological challenge, future research should take the next step in the influence of the case-mix on the MRR (for example interaction between predictors). As another strength, different strategies for dealing with missing data were checked to see if there were substantial deviations. As a limitation, the results of the present study are not completely generalizable as the included patients were characterised with ASA I-II and BMI below 35, which represent around 80% of the total THA population [20]. Patients with higher ASA classification and a higher BMI mostly score worse on the THA PROMs [33]. Adding this group to the study group of the current study will result in a more heterogeneous patient group. The mean change score will be lower and a larger CI is expected. It would be harder to comply the MRR conditions in lower response rate groups. Therefore, the MRR will be higher. Expected is that the more homogeneous the patient group is, the lower the MRR could be. Therefore, external validation of the results in a variety of hospitals settings is needed. This study was executed in a medium sized orthopaedic hospital. Another suggestion for further research is to investigate the minimum response number instead of MRR (percentage) as hospitals could be small or large in THA volume. Expected is that a combination of number and percentage is needed.

In general, PROs collection has already begun to yield results. However, there is still much work to do until significant benefits with respect to evaluating THA and improving patient care are found [34, 35]. Studies such as the present study are important, since PROs are increasingly transparent and publicly available while current validity is questionable without sufficient scientific evidence on the possible effects of (in)complete PROs collection. Health care providers, decision makers and payers are often unaware of these effects.

## Conclusions

To adequately evaluate THA procedures from a clinical perspective in theory a response rate on PROMs of 100% is needed. All stakeholders using THA PROs should be aware that 100% of the THA patients should respond on both preoperative and 3 month postoperative PROMs to measure similar change scores, to keep the influence of each change score predictor and to maintain a representative random sample of THA patients. For now, taking the first step in improving evaluation of THA for quality control, advised is to require that 60% of the THA patients should be responders on both time points as the lower limit in evaluating THA PROs.

## Supplementary information

**Additional file 1.** Additional figures and tables.

## Data Availability

The datasets used and/or analysed during the current study are available from the corresponding author on reasonable request.

## Abbreviations

ASA: American Society of Anaesthesiologists
BMI: Body mass index
CI: Confidence interval
EQ VAS: EuroQol Visual Analogue Scale
EQ-5D descriptive system: EuroQol 5 dimensions descriptive system
EQ-5D-3L: EuroQol 5 dimensions 3-level version
HOOS-PS: Hip disability and osteoarthritis outcome score-physical function short-form
ISAR: International Society of Arthroplasty Registries
MCID: Minimal clinical important difference
MRR: Minimum response rate
NOV: Dutch Orthopaedic Association
NRS: Numeric rating scale
OHS: Oxford Hip Score
PROMs: Patient-reported outcome measures
PROs: Patient-reported outcomes
THA: Total hip arthroplasty
