# Supplementary material for: What is the minimum response rate on patient-reported outcome measures needed to adequately evaluate total hip arthroplasties?
Source: Health Qual Life Outcomes. 2020 Dec 2;18:379. doi: 10.1186/s12955-020-01628-1 (PMC7709349; doi:10.1186/s12955-020-01628-1)
Supplement: Supplementary file 1 — Additional file 1. Additional figures and tables. [file 12955_2020_1628_MOESM1_ESM.docx]

**Additional file 1.** Extra figures and tables

**
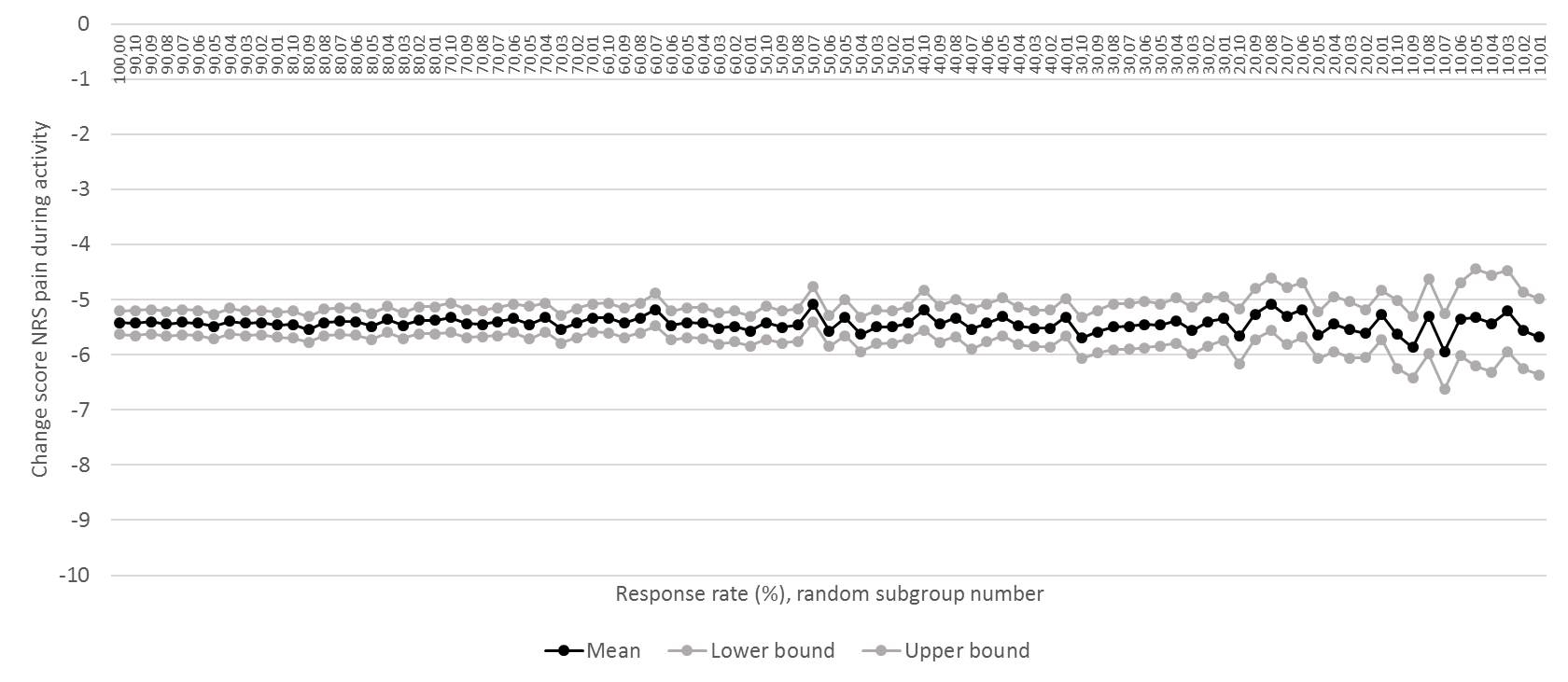
**

**Fig. 1** Mean NRS pain during activity change score per group

NRS: numeric rating scale

**Table 1** Number of NRS pain during activity groups per response rate with predictors as significant predictor or equal distribution

|  | Charnley score | | Gender | | Comorbidity | | Anxiety | | Age | | BMI | |
| --- | --- | --- | --- | --- | --- | --- | --- | --- | --- | --- | --- | --- |
|  | Significant predictor? | Equal distribution? | Significant predictor? | Equal distribution? | Significant predictor? | Equal distribution? | Significant predictor? | Equal distribution? | Significant predictor? | Equal distribution? | Significant predictor? | Equal distribution? |
| 100% (p-value) | 0.564 | x | 0.000 | x | 0.532 | x | 0.444 | x | 0.000 | x | 0.051 | x |
| 90% (n) | 0 | 10 | 10 | 10 | 0 | 10 | 0 | 10 | 10 | 10 | 5 | 10 |
| 80% (n) | 0 | 10 | 10 | 10 | 0 | 10 | 0 | 10 | 10 | 10 | 3 | 10 |
| 70% (n) | 0 | 10 | 10 | 10 | 0 | 10 | 0 | 10 | 10 | 10 | 1 | 10 |
| 60% (n) | 0 | 10 | 10 | 10 | 0 | 10 | 1 | 10 | 10 | 10 | 1 | 10 |
| 50% (n) | 1 | 10 | 9 | 10 | 0 | 10 | 1 | 10 | 7 | 10 | 3 | 10 |
| 40% (n) | 0 | 10 | 9 | 10 | 0 | 10 | 1 | 10 | 3 | 10 | 3 | 10 |
| 30% (n) | 0 | 10 | 7 | 10 | 0 | 10 | 0 | 10 | 4 | 10 | 4 | 10 |
| 20% (n) | 0 | 10 | 2 | 10 | 1 | 10 | 0 | 10 | 4 | 10 | 2 | 10 |
| 10% (n) | 0 | 9 | 0 | 10 | 1 | 10 | 0 | 10 | 2 | 10 | 1 | 10 |

BMI: body mass index; NRS: numeric rating scale


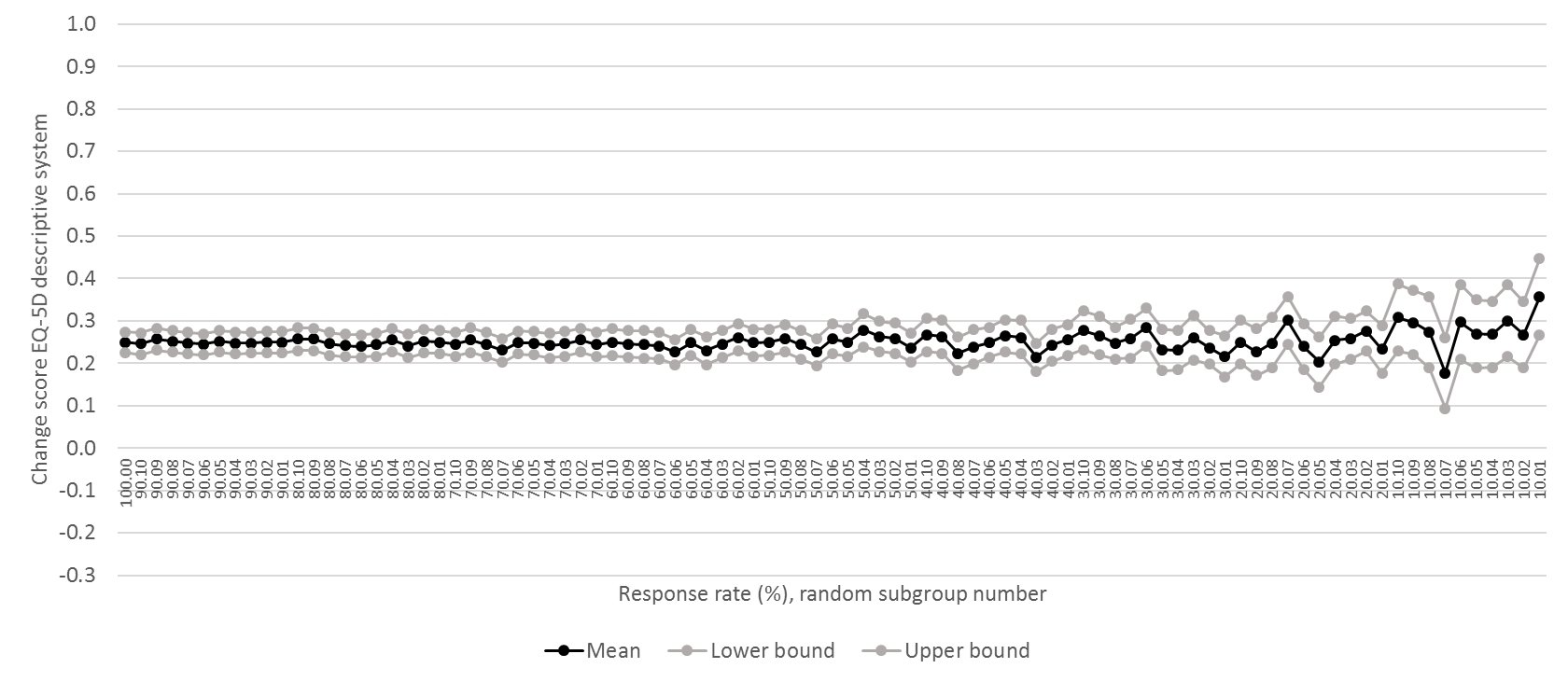


**Fig. 2** Mean change score EQ-5D descriptive system per group

EQ-5D descriptive system: EuroQol 5 dimensions descriptive system

**Table 2** Number of EQ-5D descriptive system groups per response rate with predictors as significant predictor or equal distribution

|  | Charnley score | | Gender | | Comorbidity | | Anxiety | | Age | | BMI | |
| --- | --- | --- | --- | --- | --- | --- | --- | --- | --- | --- | --- | --- |
|  | Significant predictor? | Equal distribution? | Significant predictor? | Equal distribution? | Significant predictor? | Equal distribution? | Significant predictor? | Equal distribution? | Significant predictor? | Equal distribution? | Significant predictor? | Equal distribution? |
| 100% (p-value) | 0.429 | x | 0.001 | x | 0.162 | x | 0.000 | x | 0.004 | x | 0.019 | x |
| 90% (n) | 0 | 10 | 10 | 10 | 1 | 10 | 10 | 10 | 10 | 10 | 5 | 10 |
| 80% (n) | 0 | 10 | 10 | 10 | 0 | 10 | 10 | 10 | 10 | 10 | 5 | 10 |
| 70% (n) | 0 | 10 | 10 | 10 | 1 | 10 | 10 | 10 | 6 | 10 | 5 | 10 |
| 60% (n) | 1 | 10 | 9 | 10 | 1 | 10 | 10 | 10 | 7 | 10 | 4 | 10 |
| 50% (n) | 0 | 10 | 8 | 10 | 3 | 10 | 10 | 10 | 4 | 10 | 3 | 10 |
| 40% (n) | 1 | 10 | 4 | 10 | 2 | 10 | 10 | 10 | 5 | 10 | 3 | 10 |
| 30% (n) | 1 | 10 | 6 | 10 | 0 | 10 | 10 | 10 | 5 | 10 | 3 | 10 |
| 20% (n) | 0 | 9 | 2 | 9 | 0 | 10 | 10 | 10 | 2 | 10 | 1 | 10 |
| 10% (n) | 1 | 10 | 0 | 10 | 0 | 10 | 2 | 9 | 0 | 10 | 1 | 10 |

BMI: body mass index; EQ-5D descriptive system: EuroQol 5 dimensions descriptive system; NRS: numeric rating scale


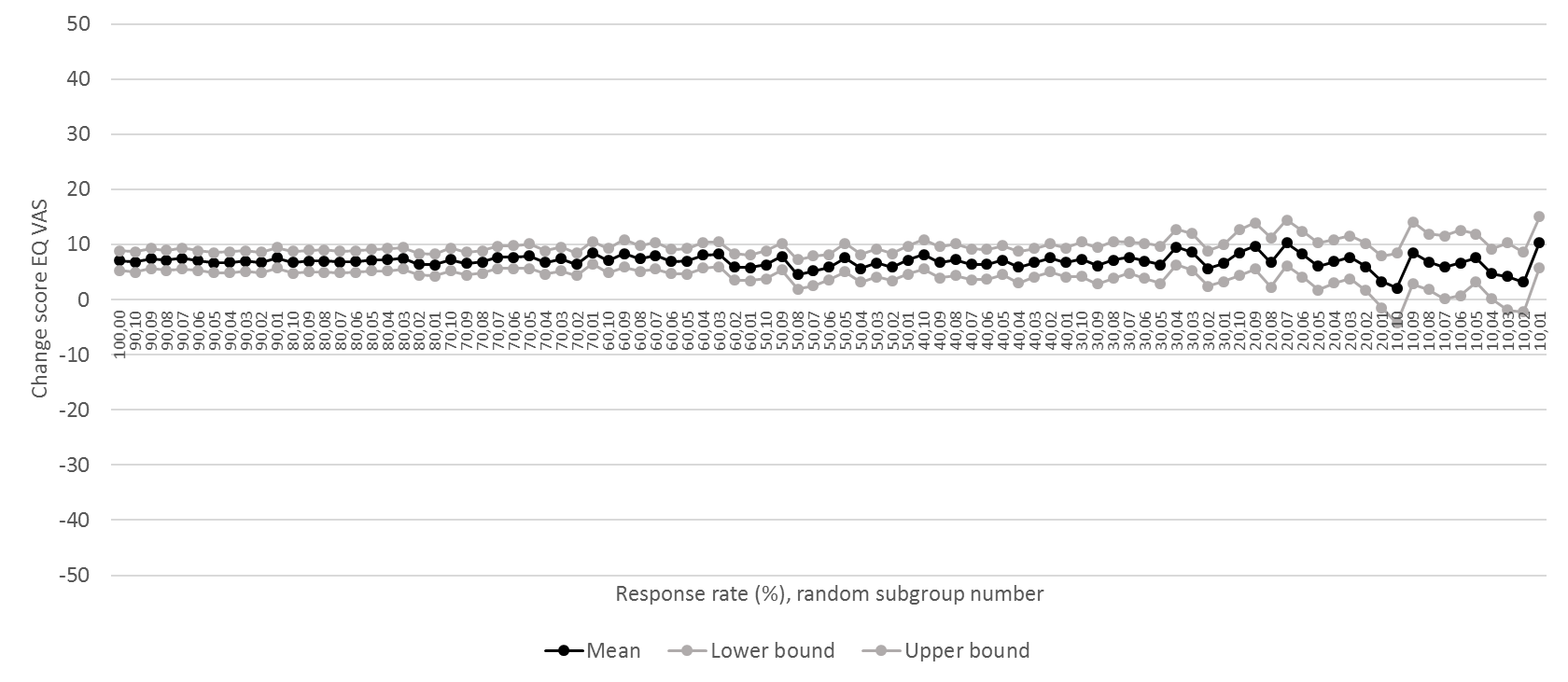


**Fig. 3** Mean EQ VAS change score per group

EQ VAS: EuroQol Visual Analogue Scale

**Table 3** Number of EQ VAS groups per response rate with predictors as significant predictor or equal distribution

|  | Charnley score | | Gender | | Comorbidity | | Anxiety | | Age | | BMI | |
| --- | --- | --- | --- | --- | --- | --- | --- | --- | --- | --- | --- | --- |
|  | Significant predictor? | Equal distribution? | Significant predictor? | Equal distribution? | Significant predictor? | Equal distribution? | Significant predictor? | Equal distribution? | Significant predictor? | Equal distribution? | Significant predictor? | Equal distribution? |
| 100% (p-value) | 0.722 | x | 0.001 | x | 0.003 | x | 0.000 | x | 0.245 | x | 0.821 | x |
| 90% (n) | 0 | 10 | 10 | 10 | 10 | 10 | 10 | 10 | 0 | 10 | 0 | 10 |
| 80% (n) | 0 | 10 | 10 | 10 | 10 | 10 | 10 | 10 | 1 | 10 | 0 | 10 |
| 70% (n) | 0 | 10 | 10 | 10 | 9 | 10 | 10 | 10 | 0 | 10 | 0 | 10 |
| 60% (n) | 0 | 10 | 7 | 10 | 9 | 9 | 10 | 10 | 1 | 10 | 0 | 10 |
| 50% (n) | 1 | 10 | 7 | 10 | 6 | 10 | 10 | 10 | 1 | 10 | 0 | 10 |
| 40% (n) | 0 | 10 | 6 | 10 | 5 | 10 | 7 | 10 | 0 | 10 | 0 | 10 |
| 30% (n) | 0 | 10 | 4 | 10 | 2 | 10 | 8 | 10 | 0 | 10 | 0 | 10 |
| 20% (n) | 0 | 10 | 2 | 10 | 5 | 10 | 4 | 9 | 0 | 10 | 0 | 10 |
| 10% (n) | 1 | 10 | 2 | 9 | 1 | 9 | 1 | 10 | 1 | 10 | 1 | 10 |

BMI: body mass index; EQ VAS: EuroQol Visual Analogue Scale; NRS: numeric rating scale

*
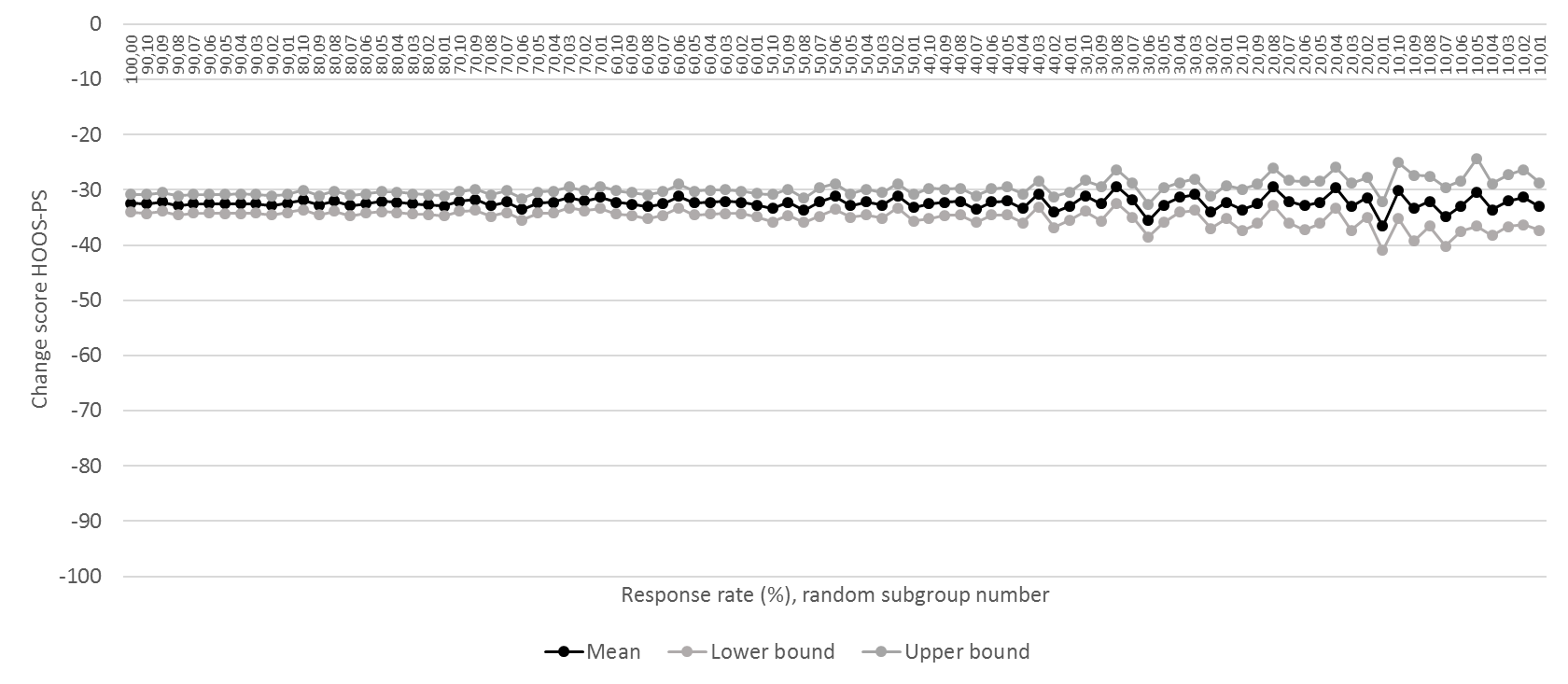
*

**Fig. 4** Mean HOOS-PS change score per group

HOOS-PS: Hip disability and Osteoarthritis Outcome Score-Physical function Short-form

**Table 4** Number of HOOS-PS groups per response rate with predictors as significant predictor or equal distribution

|  | Charnley score | | Gender | | Comorbidity | | Anxiety | | Age | | BMI | |
| --- | --- | --- | --- | --- | --- | --- | --- | --- | --- | --- | --- | --- |
|  | Significant predictor? | Equal distribution? | Significant predictor? | Equal distribution? | Significant predictor? | Equal distribution? | Significant predictor? | Equal distribution? | Significant predictor? | Equal distribution? | Significant predictor? | Equal distribution? |
| 100% (p-value) | 0.421 | x | 0.000 | x | 0.144 | x | 0.003 | x | 0.115 | x | 0.090 | x |
| 90% (n) | 0 | 10 | 10 | 10 | 0 | 10 | 10 | 10 | 0 | 10 | 1 | 10 |
| 80% (n) | 1 | 10 | 10 | 10 | 1 | 10 | 10 | 10 | 0 | 10 | 3 | 10 |
| 70% (n) | 0 | 10 | 10 | 10 | 1 | 10 | 8 | 10 | 2 | 10 | 3 | 10 |
| 60% (n) | 0 | 10 | 10 | 10 | 0 | 10 | 8 | 10 | 1 | 10 | 1 | 10 |
| 50% (n) | 3 | 10 | 7 | 10 | 1 | 10 | 6 | 10 | 0 | 10 | 0 | 10 |
| 40% (n) | 0 | 10 | 8 | 10 | 0 | 10 | 4 | 10 | 0 | 10 | 1 | 10 |
| 30% (n) | 1 | 10 | 6 | 10 | 0 | 10 | 4 | 10 | 2 | 10 | 3 | 10 |
| 20% (n) | 0 | 10 | 2 | 10 | 2 | 10 | 4 | 10 | 2 | 10 | 0 | 10 |
| 10% (n) | 0 | 10 | 0 | 9 | 1 | 9 | 2 | 10 | 0 | 10 | 0 | 10 |

BMI: body mass index; HOOS-PS: Hip disability and Osteoarthritis Outcome Score-Physical function Short-form; NRS: numeric rating scale

*
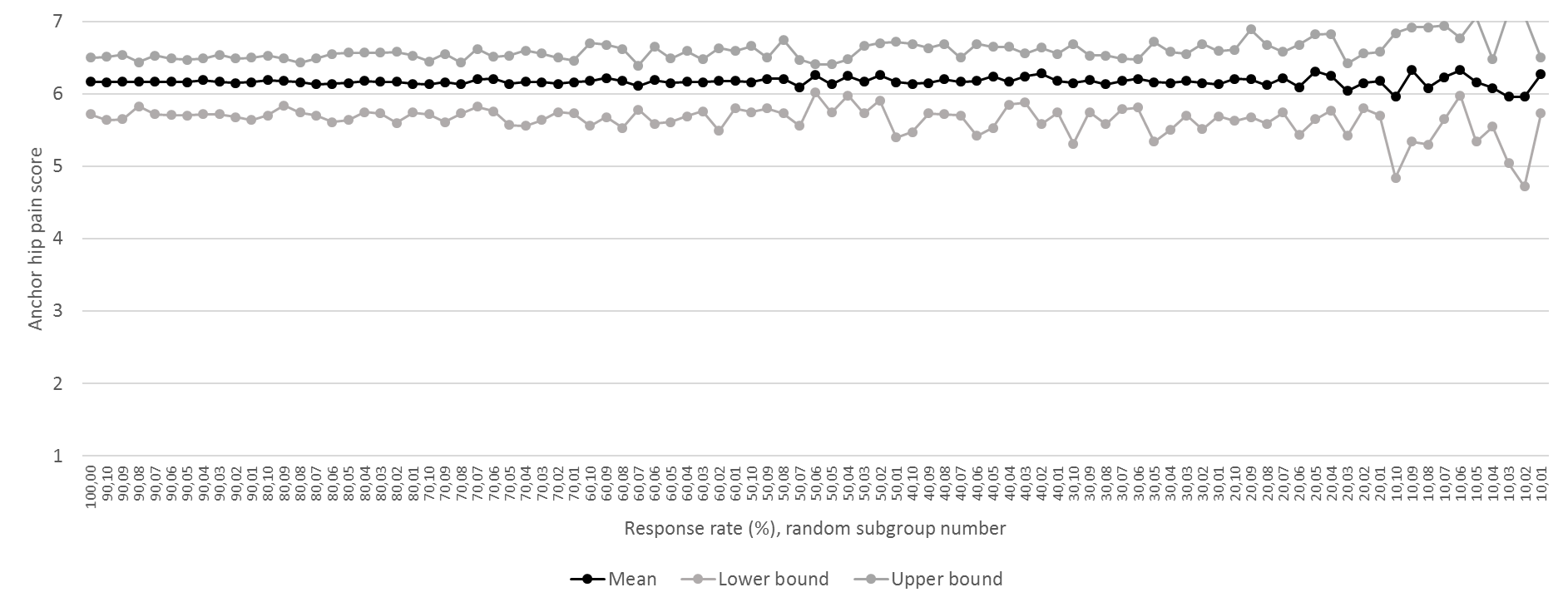
*

**Fig. 5** Mean anchor hip pain score per group

**Table 5** Number of anchor hip pain groups per response rate with predictors as significant predictor or equal distribution

|  | Charnley score | | Gender | | Comorbidity | | Anxiety | | Age | | BMI | |
| --- | --- | --- | --- | --- | --- | --- | --- | --- | --- | --- | --- | --- |
|  | Significant predictor? | Equal distribution? | Significant predictor? | Equal distribution? | Significant predictor? | Equal distribution? | Significant predictor? | Equal distribution? | Significant predictor? | Equal distribution? | Significant predictor? | Equal distribution? |
| 100% (p-value) | 0.595 | x | 0.040 | x | 0.022 | x | 0.156 | x | 0.514 | x | 0.350 | x |
| 90% (n) | 0 | 10 | 2 | 10 | 6 | 10 | 1 | 10 | 0 | 10 | 0 | 10 |
| 80% (n) | 0 | 10 | 5 | 10 | 4 | 10 | 1 | 10 | 0 | 10 | 0 | 10 |
| 70% (n) | 0 | 10 | 3 | 10 | 5 | 10 | 0 | 10 | 0 | 10 | 0 | 10 |
| 60% (n) | 0 | 10 | 1 | 10 | 6 | 10 | 1 | 10 | 0 | 10 | 0 | 10 |
| 50% (n) | 0 | 10 | 1 | 10 | 2 | 10 | 0 | 10 | 0 | 10 | 1 | 10 |
| 40% (n) | 0 | 10 | 2 | 10 | 1 | 10 | 2 | 10 | 0 | 10 | 2 | 10 |
| 30% (n) | 0 | 10 | 0 | 10 | 1 | 10 | 0 | 10 | 0 | 10 | 2 | 10 |
| 20% (n) | 0 | 10 | 1 | 10 | 1 | 10 | 0 | 10 | 0 | 10 | 0 | 10 |
| 10% (n) | 0 | 9 | 0 | 10 | 4 | 10 | 0 | 9 | 0 | 10 | 2 | 9 |

BMI: body mass index; NRS: numeric rating scale


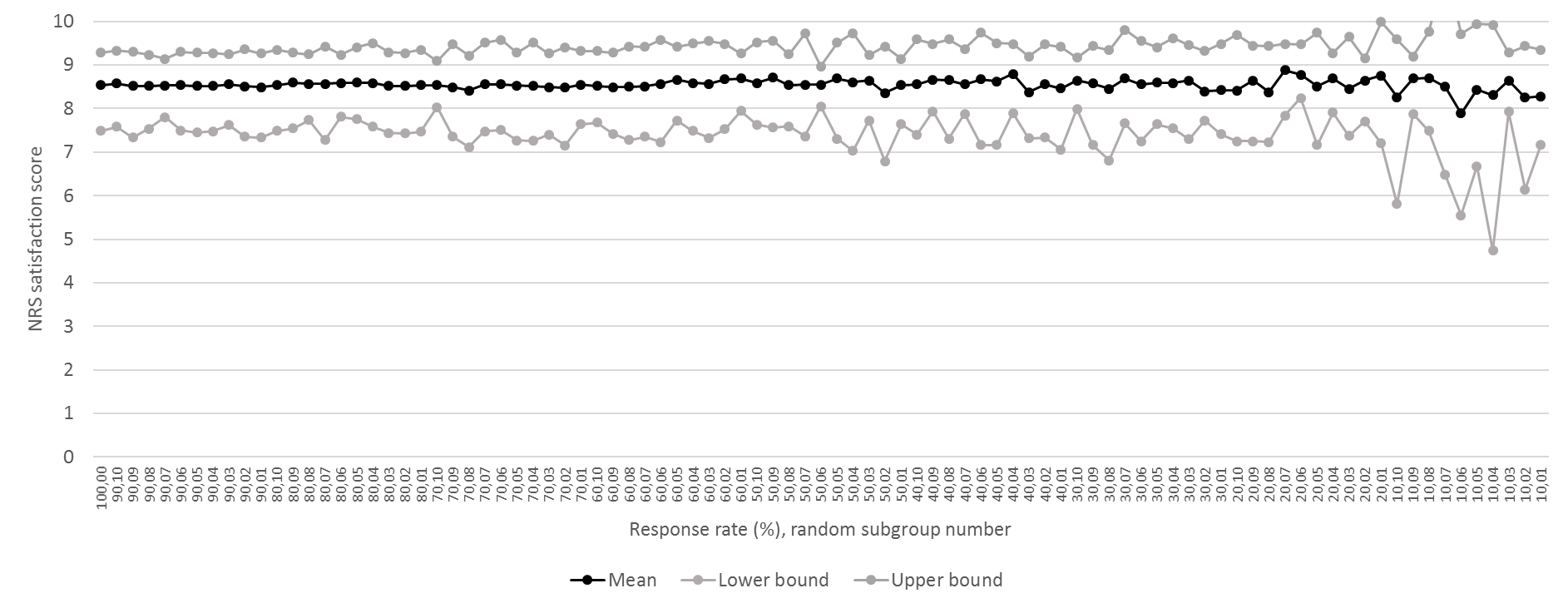


**Fig. 6** Mean NRS satisfaction score per group

NRS: numeric rating scale

**Table 6** Number of NRS satisfaction groups per response rate with predictors as significant predictor or equal distribution

|  | Charnley score | | Gender | | Comorbidity | | Anxiety | | Age | | BMI | |
| --- | --- | --- | --- | --- | --- | --- | --- | --- | --- | --- | --- | --- |
|  | Significant predictor? | Equal distribution? | Significant predictor? | Equal distribution? | Significant predictor? | Equal distribution? | Significant predictor? | Equal distribution? | Significant predictor? | Equal distribution? | Significant predictor? | Equal distribution? |
| 100% (p-value) | 0.787 | x | 0.013 | x | 0.184 | x | 0.075 | x | 0.734 | x | 0.029 | x |
| 90% (n) | 0 | 10 | 10 | 10 | 0 | 10 | 1 | 10 | 0 | 10 | 4 | 10 |
| 80% (n) | 0 | 10 | 7 | 10 | 1 | 10 | 2 | 10 | 0 | 10 | 5 | 10 |
| 70% (n) | 0 | 10 | 5 | 10 | 1 | 10 | 1 | 10 | 0 | 10 | 4 | 10 |
| 60% (n) | 0 | 10 | 7 | 10 | 2 | 10 | 1 | 10 | 0 | 10 | 4 | 10 |
| 50% (n) | 0 | 10 | 3 | 10 | 1 | 10 | 2 | 10 | 0 | 10 | 3 | 10 |
| 40% (n) | 0 | 10 | 3 | 10 | 1 | 10 | 0 | 10 | 1 | 10 | 3 | 10 |
| 30% (n) | 2 | 10 | 3 | 10 | 0 | 10 | 2 | 10 | 0 | 10 | 0 | 10 |
| 20% (n) | 0 | 10 | 1 | 10 | 0 | 8 | 0 | 10 | 3 | 10 | 0 | 10 |
| 10% (n) | 0 | 9 | 0 | 9 | 0 | 9 | 1 | 9 | 1 | 10 | 0 | 10 |

BMI: body mass index; NRS: numeric rating scale
